# Automated Behavioral Coding to Enhance the Effectiveness of Motivational Interviewing in a Chat-Based Suicide Prevention Helpline: Secondary Analysis of a Clinical Trial

**DOI:** 10.2196/53562

**Published:** 2024-08-01

**Authors:** Mathijs Pellemans, Salim Salmi, Saskia Mérelle, Wilco Janssen, Rob van der Mei

**Affiliations:** 1 Department of Mathematics Vrije Universiteit Amsterdam Amsterdam Netherlands; 2 113 Suicide Prevention Amsterdam Netherlands; 3 Centrum Wiskunde & Informatica Amsterdam Netherlands; 4 Department of Psychiatry Amsterdam UMC Amsterdam Netherlands

**Keywords:** motivational interviewing, behavioral coding, suicide prevention, artificial intelligence, effectiveness, counseling, support tool, online help, mental health

## Abstract

**Background:**

With the rise of computer science and artificial intelligence, analyzing large data sets promises enormous potential in gaining insights for developing and improving evidence-based health interventions. One such intervention is the counseling strategy motivational interviewing (MI), which has been found effective in improving a wide range of health-related behaviors. Despite the simplicity of its principles, MI can be a challenging skill to learn and requires expertise to apply effectively.

**Objective:**

This study aims to investigate the performance of artificial intelligence models in classifying MI behavior and explore the feasibility of using these models in online helplines for mental health as an automated support tool for counselors in clinical practice.

**Methods:**

We used a coded data set of 253 MI counseling chat sessions from the 113 Suicide Prevention helpline. With 23,982 messages coded with the MI Sequential Code for Observing Process Exchanges codebook, we trained and evaluated 4 machine learning models and 1 deep learning model to classify client- and counselor MI behavior based on language use.

**Results:**

The deep learning model BERTje outperformed all machine learning models, accurately predicting counselor behavior (accuracy=0.72, area under the curve [AUC]=0.95, Cohen κ=0.69). It differentiated MI congruent and incongruent counselor behavior (AUC=0.92, κ=0.65) and evocative and nonevocative language (AUC=0.92, κ=0.66). For client behavior, the model achieved an accuracy of 0.70 (AUC=0.89, κ=0.55). The model’s interpretable predictions discerned client change talk and sustain talk, counselor affirmations, and reflection types, facilitating valuable counselor feedback.

**Conclusions:**

The results of this study demonstrate that artificial intelligence techniques can accurately classify MI behavior, indicating their potential as a valuable tool for enhancing MI proficiency in online helplines for mental health. Provided that the data set size is sufficiently large with enough training samples for each behavioral code, these methods can be trained and applied to other domains and languages, offering a scalable and cost-effective way to evaluate MI adherence, accelerate behavioral coding, and provide therapists with personalized, quick, and objective feedback.

## Introduction

### Background

Motivational interviewing (MI) is a client-centered counseling style that helps individuals change their behavior by resolving ambivalence using nondirective conversation techniques. It has been found effective in improving a wide range of health-related behaviors [1], such as weight management [2], addictive behaviors [3], and promoting self-management in patients with chronic health conditions [4]. During MI, counselors use a specific set of conversation techniques, most notably open-ended questions, and reflections, to let clients voice their own arguments for a particular behavior change. They are encouraged to elaborate on these reasons. This way, clients reason themselves into changing their behavior, strengthening their intrinsic motivation for the behavior change and avoiding the often-triggered defensive mechanisms when others argue for such a change. MI is a crucial anchor in guiding the counseling process during chat-based conversations at the Dutch national organization for suicide prevention (Dutch: 113 Zelfmoordpreventie).

Despite the simplicity of its principles, MI can be a challenging skill to learn, and MI requires substantial expertise to apply effectively [5]. Earlier research has shown that counselors at 113 applied MI techniques consistently during chat conversations but could not strategically deploy MI techniques to elicit enough change talk from clients to change their behavior intrinsically [6]. Therefore, it becomes imperative to improve the proficiency level of counselors in applying MI techniques to conduct conversations more effectively, especially, since eliciting change talk from clients’ accounts for the effectiveness of MI [7].

One way to achieve this is through the automated evaluation of counselor responses to clients’ expressed language utterances. By increasing their behavior awareness, counselors can significantly reduce cognitive effort and reflect on MI insights for education. Multiple validated proficiency measures exist for MI [8], and tools are already in development to measure treatment fidelity automatically [5,9]. In the context of chat-based helplines, these tools can provide counselors with immediate feedback during ongoing chats, potentially improving the quality of the service. Chat-based helplines also present a unique opportunity for developing such treatment fidelity tools due to the availability of extensive databases of written conversations. Also, Lundahl et al [1] found that MI is a robust intervention across patient characteristics, which gives these tools broad applicability in numerous health settings.

### Enhancing MI Effectiveness Through Artificial Intelligence

Artificial intelligence (AI) has made a significant impact in recent years in many fields, the field of clinical mental health being no exception. AI offers enormous potential to analyze large data sets through machine learning (ML) algorithms. By analyzing data from MI sessions, ML algorithms can identify successful and unsuccessful applications of MI concepts, supporting and training MI practitioners. In addition, counselors can use an AI support tool to evaluate the quality of their sessions. These tools can help improve and assess counselors’ MI proficiency cost-effectively and tailor additional training to their needs.

AI can also speed up the coding of MI sessions, making it easier to analyze and provide feedback *during* and *after* a counseling session. Providing counselors with ongoing feedback seemed especially important for learning MI [10]. Besides, immediate feedback has a more powerful impact on skill development than delayed feedback [9].

Although large amounts of data are typically required to train ML models to perform well on complex tasks such as capturing MI behavior, AI has developed techniques that perform well in domains with limited available data, providing insights into developing and improving evidence-based health interventions [11].

Since behavioral coding is often time-consuming, several studies have explored the automated annotation of MI transcripts in counseling sessions using ML techniques. Hasan et al [12] conducted experiments on automating the annotation of weight loss counseling sessions using the MI Sequential Code for Observing Process Exchanges (MI-SCOPE) codebook. They assessed various classification methods, incorporating linguistic, contextual, and semantic features based on linguistic inquiry and word count (LIWC) [13]. Their experiments showed that a support vector machine (SVM) model with these features achieved 75% accuracy in automatically annotating MI transcripts containing 17 behavioral codes. Idalski Carcone et al [14] aimed to develop a classification model to automatically code clinical encounter transcripts about weight loss using the MI-SCOPE behavioral code scheme. Their SVM model achieved a 69.6% *F*_1_-score on 17 classes. Tanana et al [15] introduced 2 ML models for automatically coding MI sessions. The researchers found that the best-performing ML model had a good or higher utterance–level agreement with human coders (Cohen κ>0.60) for open and closed questions, affirmations, and giving information. However, there was a poor agreement for client change talk, client sustain talk, and therapist MI-congruent behaviors. Pérez-Rosas et al [16] presented a model for predicting MI counselor behaviors in multiple medical settings. Their SVM classifier performed well for more frequently encountered behaviors (reflections and questions) using N-grams, syntactic, and semantic LIWC features [13]. However, the performance varied much per predicted class, also obtaining lower performance for emphasizing autonomy and affirmations. Tavabi et al [17] compared the classification performance of client behaviors throughout MI psychotherapy sessions with students having alcohol-related problems using pretrained embeddings and interpretable LIWC features. Their best-performing model (pretrained RoBERTa) achieved an *F*_1_-score of 0.66 in a 3-class classification. Saiyed et al [5] developed a Technology-Assisted Motivational Interviewing Coach incorporating ML models to deliver MI predictions for counseling sessions about tobacco cessation. Using a novel deep learning architecture combining a large fine-tuned language model and graph theory, the automated change talk/sustain talk/follow-neutral classifier achieved an accuracy of 0.74 and an *F*_1_-score of 0.75.

For a comprehensive overview of research papers using ML to classify MI behavior for assessing treatment fidelity, we refer to Ahmadi et al [18]. Multimedia Appendix 1 provides a schematic overview of the related work, including study context, study size, used fidelity measure, and—if reported—the coding reliability estimate. The application domain significantly varies, which also applies to the reporting of coding reliability estimates. Assessing treatment fidelity and reliability holds enormous relevance for evaluating study quality and the successful integration of MI into practice. A meta-analysis on the effect of MI on medication adherence found that interventions that examined fidelity and provided counselors with feedback on their fidelity were more effective than those that did not [19], indicating that a higher fidelity may lead to improved intervention outcomes. Frost et al [20] highlighted that fidelity is often poorly measured and reported. Moreover, MI adherence and fidelity demonstrated considerable variation across different settings and application domains [20-22].

Despite the promising algorithm performances, predicting MI-congruent counselor behavior and eliciting client change talk was challenging [15,16]. Besides, few studies adhered to best-practice ML guidelines. Although testing methods on unseen data is an essential measure of method performance in ML, only a small proportion of studies tested their methods on holdout data. A holdout subset provides a final estimate of the ML model’s performance after it has been trained and validated. Similarly, Ahmadi et al [18] found that almost half of the studies in their review did not describe how they undertook data preprocessing.

For readers to assume that ML methods will generalize on future data, researchers must report these methodological processes clearly and transparently, including robust coding reliability and fidelity measures. Previous studies showed the feasibility of providing feedback to counselors via a support tool [23], consistently measuring fidelity and reporting Krippendorff’s alpha estimates for interrater reliability.

### This Study

This study aims to investigate the performance of AI models in classifying MI behavior and explore the feasibility of using these models in helplines as an automated support tool for counselors in clinical practice. We use a coded data set of 253 chat-based MI counseling sessions conducted at the chat helpline of 113 Suicide Prevention. We train and compare different AI algorithms to classify client- and counselor MI behavior based on language use to identify the most suitable model for the task.

The key contributions of this paper are as follows: (1) to the best of our knowledge, this is the first research that combines AI and MI with a focus on suicide prevention. (2) We aim to assist counselors in a suicide prevention helpline to overcome the practical challenges of eliciting change talk and enhancing awareness of conversation quality by providing feedback. (3) Our AI approach is described in detail, adhering to the best practices in the field and establishing a benchmark for implementing similar techniques in various settings.

## Methods

### Data Set

This study used a coded data set of 253 chat conversations (constituting 23,982 chat messages, 12,125 counselor messages, and 11,857 client messages) from chat-based MI counseling sessions conducted at 113 between July 2020 and January 2021. All chats were Dutch language chats and lasted at least 20 minutes. Janssen et al [6] described the exact data collection procedure.

### Participants

Participants in the data set contacted the 113 crisis chat service in the Netherlands between 8:30 AM and 10:30 PM. All clients who spoke Dutch, filled out both a pre-and postchat questionnaire, and reported at least some suicidal ideation on the prechat questionnaire (score ≥1 on a 7-point Likert scale) were eligible for participation in the study [6].

### Ethical Considerations

The ethics review committee of the VU University Medical Center in Amsterdam reviewed and approved this study (2020.105). The national legislation and institutional requirements did not require written informed consent from the participants. All nonessential identifying details have been omitted.

For analysis, we used only (cleaned) text of the chat messages without any personalized metainformation (including—but not limited to—age, gender, ethnicity, or clinical diagnosis). There was no collection procedure for other additional data *before*, *during*, or *after* a chat conversation. The publication of the results did not have any negative impact on the participants. Participants did not receive any form of compensation.

### Procedure

#### Measures

Practical instruments exist to understand the quality and effectiveness of applying MI in counseling conversations. Researchers coded the data set with the MI-SCOPE coding instrument [24]. Researchers created this tool to explore the relationships between essential theoretical constructs of MI, the therapy process, and client outcomes. The focus is on analyzing the relationship between MI-specific interviewer behaviors and subsequent client behaviors within an MI session. The MI-SCOPE combines 2 successful coding systems: the MISC [25] and the commitment language coding system developed by Amrhein et al [26].

The MI-SCOPE provides 5 indices of treatment integrity, including the percentage of MI-consistent responses, the relative amount of open questions, the proportion of complex reflections, the reflection-to-question ratio, and the proportion of change talk. Hurlocker et al [8] indicated that reliability estimates for the MI-SCOPE are generally fair to excellent.

While most studies have used the MISC only [18] or the well-validated but relatively short MITI, these instruments do not provide information on the amount of change talk and sustain talk expressed by the client, whereas the MI-SCOPE does. The MI-SCOPE thus covers more aspects of MI, incorporating both client and counselor behavior, and is more time-efficient [27]. Had the MITI or the MISC been used instead of the MI-SCOPE, Janssen et al [6] would not have detected the insufficiency of MI effectiveness in eliciting client change talk. Research by Magill et al [28] and Pas et al [29] also emphasizes the importance of fidelity measures in MI. The studies suggest that therapist adherence to MI techniques can influence client engagement and outcomes, and high-fidelity counseling can improve intervention effectiveness.

#### Data Set Coding and Reliability

Researchers who coded the data set [6] followed recommendations by O’Connor and Joffe [30] and described the exact coding procedure in their paper. Of the total number of counselor messages, Janssen et al [6] labeled 9177 counselor messages with fine-grained MI behavioral codes and 2948 chat messages with less fine-grained codes, indicating only MI congruency.

The coding process for all MI conversations lasted 4 months, from January 1, 2021, to May 12, 2021. The researchers used the qualitative data analysis tool ATLAS.ti 9 for the coding procedure and assessing reliability estimates. Intercoder reliability was sufficient, as Janssen et al [6] reported a Krippendorff’s alpha-binary of 0.82 for the percentage of MI-consistent responses and 0.90 or higher for open questions, closed questions, and reflections. Generally, researchers consider an alpha-binary over 0.90 acceptable in all cases, while an alpha-binary ranging from 0.80 to 0.90 is deemed sufficient.

#### Code Grouping

To predict counselor behavior congruent with MI, we partnered with a seasoned psychologist at 113 (listed as the fourth author, WJ) to group the annotated MI behavioral codes—as outlined in the MI-SCOPE coding manual [31]—considering the practical challenges within the counseling process.

We combined all closed questions, negative and neutral reflections, and positive reflections (simple and complex), yielding 17 code groups for counselor language. For counselor language, we created 2 groups of the MI-SCOPE codes based on whether counselor language elicited client change talk (7477 nonevocative messages; 1700 evocative messages) and whether it was MI congruent (8765 MI-congruent messages; 3360 MI incongruent messages; see Table 1). We excluded the labels *Raise Concern* and *Direct* from further analysis due to their low occurrence in the data set. We did not assign detailed labels to the 4 client codes (*Ask, Follow/Neutral, Change Talk, and Sustain Talk*). Initial data analysis revealed that only 18.52% of all counselor messages were evocative and 70.33% were MI congruent.

**Table 1:** MI^a^ code groups for counselor language and whether or not a code is assigned evocative or MI congruent.

MI code group	Evocative	MI congruent
Advise with Permission	*×*	✓
Advise without Permission	*×*	*×*
Affirm (Aff)	✓	✓
Closed Question	*×*	*×*
Confront (Con)	*×*	*×*
Emphasize Control (Econ)	✓	✓
Filler (Fill)	*×*	*×*
General Information (GI)	*×*	*×*
Open Question (OQ+)	✓	✓
Open Question (OQ*−*)	*×*	✓
Open Question (OQ0)	*×*	✓
Permission Seeking	*×*	✓
Reflection (+)	✓	✓
Reflection (0*−*)	*×*	✓
Self-Disclose (Sdis)	*×*	*×*
Structure (Str)	*×*	✓
Support (Sup)	*×*	✓

^a^MI: motivational interviewing.

### Analytic Strategy

We trained and evaluated 4 ML models and 1 deep learning model to classify client and counselor MI behavior based on language use. ML models benefit from human-extracted features, while deep learning models learn complex patterns without feature selection. Although deep learning models have better performance potential, these models require more data and have less interpretable reasoning. We further describe the feature selection process, the models, and how we addressed these limitations of the deep learning model.

#### Feature Selection

##### Available Features

In total, we extracted 5850 features for each of the MI-coded chat messages. These features included high-level concepts such as topic, grammar, and sentiment, as well as low-level concepts such as counting the occurrence of each word. For a complete list of all feature categories used for the ML models, see Multimedia Appendix 1.

##### Feature Subsets

To gain insight into the impact of each feature category on the classification performance, we created subsets by adding 1 or more feature categories to the previous subset, resulting in 8 sets of features that we used to train the ML models, starting with the initial subset containing only the basic feature categories and ending with the final set containing all extracted features.

#### Train-Validation-Test Split

For each classification problem, we split each group of chat messages—stratified by class distribution—80%:10%:10% to create training, validation, and test data sets. To rightly measure model performance, it is essential to hold out data. We used the training set exclusively to train the models, evaluated the training progress on the validation set, and obtained the final performance using the test set. Table S3 in Multimedia Appendix 1 shows the number of classes and instances for each classification problem.

#### Learning Algorithms

We trained and evaluated 4 different ML models and 1 deep learning model for each classification problem: a random forest, an SVM, k-nearest neighbors, a decision tree, and a *pretrained transformer model*.

We chose a pretrained transformer model to overcome the limitations of regular deep learning model architectures. Transformer models are a type of deep learning network that can be pretrained on a large amount of data and then fine-tuned on a smaller, more specific data set to make predictions. By pretraining on a large data set, the model can learn to understand the structure and patterns of language, making it easier to adapt to new domains, which enables the training of complex models with limited data. Researchers showed that the BERT model [32] suits this approach particularly. We used a variant of BERT (Bidirectional Encoder Representations from Transformers), called BERTje (monolingual Dutch BERT), which already has been pretrained on a large Dutch text corpus [33], and fine-tuned BERTje on our domain-specific data set for each classification problem.

A grid search technique was used to select the best model parameters, as initial testing showed that the parameter values could severely impact model performance. Table S4 in Multimedia Appendix 1 provides an overview of the models and the considered parameters. The final analysis excluded compensating for the imbalance of the class labels in the data, as initial testing also showed that it did not lead to differences in the results. To account for this, we evaluated the models using statistics that can take class imbalances into account.

We used 5-fold cross-validation to validate the models and applied minimum-maximum scaling before training. We implemented all ML models in *Python 3.8* (developed by Python Software Foundation) and implemented the fine-tuning of BERTje using *PyTorch Lightning* (developed by Lightning AI).

#### Evaluation Metrics

Computing the confusion matrix and conducting an area under the receiver operating characteristic curve analysis allowed us to assess the classifiers and obtain visual and statistical insights into their predictive performance. We also quantified the kappa statistic and accuracy for the best-performing models. We extracted the probability distribution of the predictions from all classifiers to compute the sample average *F*_1_-score. The probability distribution indicates the confidence or likelihood of a specific model prediction. For a detailed explanation of these evaluation metrics, see the study by Zheng [34] and Figures S1 and S2 in Multimedia Appendix 1 [35,36].

#### Baseline

The baseline score provides a required point of comparison when evaluating all predictive algorithms for a classification task. We consider predicting the majority class as a baseline, meaning that we select the prediction class with the most observations and use it as the outcome for all predictions. We expect the predictive models that learn from the data to perform substantially better.

#### Validity

We used identical statistics, training, validation, and test samples to evaluate the trained models, making the validation of the results comparable across all models.

#### Explainability

To interpret the output of the models, we used Shapley Additive Explanations (SHAP) [37] as a method. SHAP provides a way to obtain the contribution of each feature in the model’s prediction for a particular input. Values provided by SHAP represent a feature’s average marginal contribution toward the difference between the predicted output and the model’s expected output. A higher value indicates a higher contribution to the output and interprets it as a more important feature.

## Results

### Algorithm Performance

In this section, we present a comprehensive evaluation and performance analysis of the ML models and the transformer model BERTje across all 4 classification tasks. We further interpret the model predictions by deploying SHAP and laying out the most occurring word combinations for each prediction class.

#### Classifying Counselor Behavior

##### Fine-Grained Predictions

**Figure 1:**
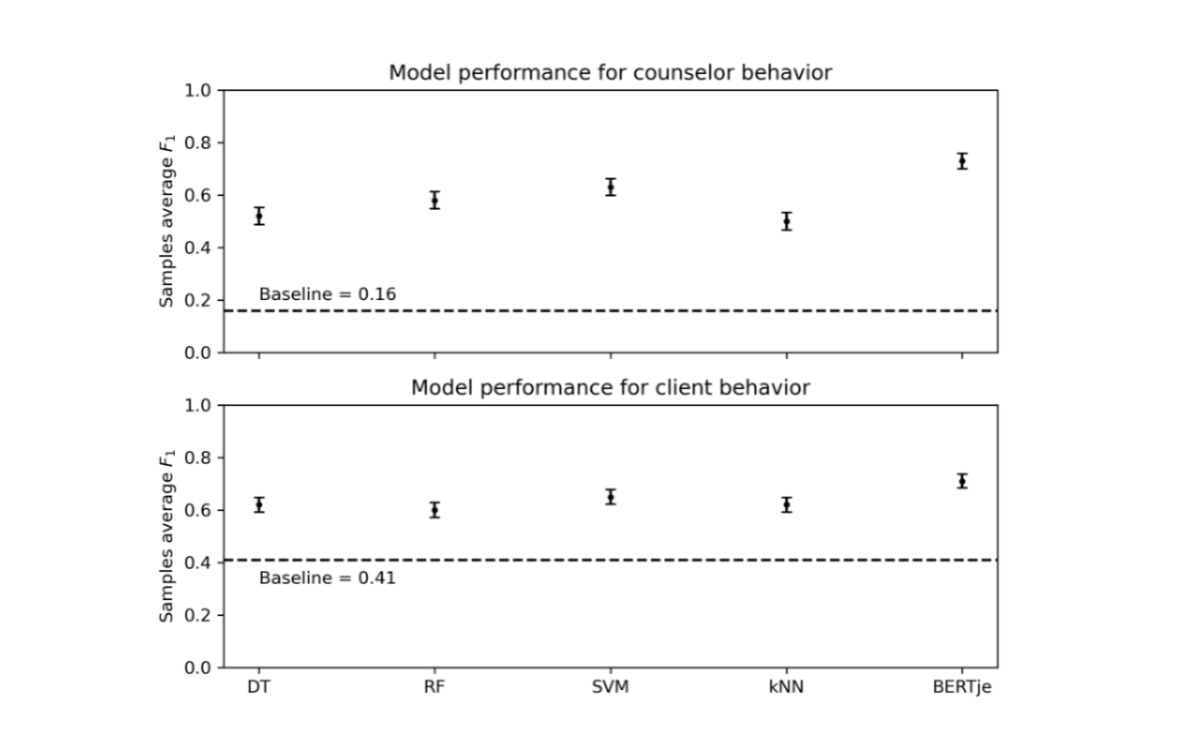
Learning algorithm performance for predicting client- and counselor behavior. The SD for the results yields 0.015 for counselor behavior and 0.013 for client behavior. CIs for the results are given by performance value ± 2 × SD. DT: decision tree; kNN: k-nearest neighbors; RF; random forest; SVM: support vector machine.

Figure 1 presents a performance comparison of the learning algorithms using the best parameters for classifying counselor behavior. The reported scores represent the average of 5 repeated runs for each model. The SVM model (*γ*=0*.*1, *C*=10) showed the highest *F*_1_-score of 0.63 among all ML models. Random forest and SVM models outperformed the decision tree and k-nearest neighbors. For an overview of the ML model performances on the different feature subsets, see Table S5 in Multimedia Appendix 1.

Incorporating textual information and word-embedding features resulted in the highest increase in the performance of the ML algorithms. Among all models tested, the transformer model BERTje achieved the highest performance with an *F*_1_-score of 0.73. Table 2 shows a detailed model performance evaluation of BERTje. With an accuracy of 0.72, kappa statistic of 0.69, and area under the curve (AUC) score of 0.95, its results represent a 350% improvement in accuracy from the baseline. The AUC scores per class ranged from 0.89 to 0.99. The model performed best on fillers and affirmations with an AUC score of 0.99 and lowest on advise without permission and confront with an AUC score of 0.89. Errors mainly occurred when predicting neutral, open questions as positive open questions (14 errors), positive reflections as neutral or negative reflections, and open questions as closed questions (17 errors).

**Table 2:** BERTje: detailed model performance evaluation on all classification tasks^a^.

Classification task			*F*_1_-score		AUC^b^	Sample average *F*_1_-score		Microaverage AUC		Macroaverage AUC		Accuracy		Kappa values
**Counselor behavior**						0.73		0.96		0.95		0.72		0.69
	Advise with Permission (AWP)	0*.*50		0*.*94										
	Advise without Permission (ADW)	0*.*39		0*.*89										
	Affirm (Aff)	0*.*86		0*.*99										
	Closed Question	0*.*85		0*.*97										
	Confront (Con)	0*.*31		0*.*89										
	Emphasize Control (Econ)	0*.*38		0*.*91										
	Filler (Fill)	0*.*80		0*.*99										
	General Information (GI)	0*.*67		0*.*96										
	Open Question (OQ+)	0*.*64		0*.*95										
	Open Question (OQ*−*)	0*.*69		0*.*97										
	Open Question (OQ0)	0*.*72		0*.*94										
	Permission Seeking (Perm)	0*.*71		0*.*96										
	Reflection (+)	0*.*51		0*.*93										
	Reflection (0*−*)	0*.*76		0*.*97										
	Self-Disclose (Sdis)	0*.*57		0*.*96										
	Structure (Str)	0*.*84		0*.*97										
	Support (Sup)	0*.*69		0*.*95										
**MI^c^ congruency**						0.88		0.94		0.92		0.87		0.65
	MI-Congruent (X MI+)	0*.*91		0*.*92										
	MI-Incongruent (X MI*−*)	0*.*76		0*.*92										
**Evocative language**						0.90		0.96		0.92		0.90		0.66
	Evocative	0*.*73		0*.*92										
	Nonevocative	0*.*94		0*.*92										
**Client behavior**						0.71		0.90		0.89		0.70		0.55
	Ask	0*.*81		0*.*99										
	Follow/Neutral (FN)	0*.*61		0*.*81										
	Change Talk (X Csa+)	0*.*66		0*.*87										
	Sustain Talk (X Csa*−*)	0*.*74		0*.*89										

^a^Cells with no numerical value indicate “not applicable.”

^b^AUC: area under the curve.

^c^MI: motivational interviewing.

##### MI Congruency

The fine-tuned BERTje model achieved a sample average *F*_1_-score of 0.88 in accurately predicting counselor behavior as either MI-congruent or MI-incongruent (see Table 2). In addition, it demonstrated a high accuracy of 0.87, accompanied by a kappa value of 0.65 and a macroaverage AUC score of 0.92. These results signify an accuracy improvement of 20.8% compared with the baseline performance (accuracy=0.72).

##### Evocative Language

The fine-tuned BERTje model achieved a sample average *F*_1_-score of 0.90 in accurately predicting whether counselor language is evocative or nonevocative (see Table 2). Moreover, it demonstrated an accuracy of 0.90, a kappa value of 0.65, and a macroaverage AUC score of 0.92. These results signify an accuracy improvement of 9.8% compared with the baseline performance (accuracy=0.82).

#### Classifying Client Behavior

Figure 1 also shows a performance comparison of the learning algorithms using the best parameters for classifying client behavior. For an overview of the ML model performances on the different feature subsets, see Table S6 in Multimedia Appendix 1. All models improved classification performance compared with the baseline. The best ML model was an SVM model (*γ*=0*.*1, *C*=10), reaching a sample average *F*_1_-score of 0.65. BERTje outperformed all ML algorithms, reaching a sample average *F*_1_-score of 0.71 with an accuracy of 0.70, Cohen κ=0.55, and a macroaverage AUC score of 0.89. These results indicate an accuracy improvement of 70.7% compared with the baseline. The AUC scores per class range from 0.81 to 0.99 (see Table 2). Although the lowest occurrence across the client messages, BERTje predicted the code *Ask* best (AUC=0.99). Follow/Neutral was the hardest to predict (AUC score=0.81). We observed that errors mainly occurred in predicting Follow/Neutral messages as commitment language.

### Feature Contributions

According to the SHAP feature importance analysis conducted on the best-performing ML models, word-embedding features held significant dominance. Furthermore, the number of question marks in a message emerged as a consistently influential factor for client- and counselor behaviors. Table S7 in Multimedia Appendix 1 shows the features that contribute most to the predictions of each class individually. Moreover, this table shows the top word combinations reflecting the language character of different client and counselor behaviors. The inferred prediction classes associated with the MI-SCOPE codes are generally interpretable. For example, client ambivalence becomes clear when counselors use reflections with word combinations such as “on one side,” “on the other side,” and “conflicted.” Concerning client commitment language, negative sentiment, and negations contributed to both sustain talk and change talk. When these features were *present*, client language was more likely to be associated with sustain talk rather than change talk. Contrarily, the *absence* of these features indicates more association with client change talk.

## Discussion

### Interpretation of the Results

The results of this study demonstrate the potential of AI models, particularly the transformer model BERTje, in classifying MI behavior in online mental health helplines. BERTje outperformed all ML models tested, achieving high levels of accuracy across all classification tasks. Although ML models obtained lower performance than the BERTje model, their high explainability adds value for gaining a deeper understanding of language use concerning specific MI behaviors.

The successful application of a fine-tuned transformer model in classifying MI behavior is consistent with other recent studies, such as by Saiyed et al [5] and Tavabi et al [17], who also used a fine-tuned transformer model to classify MI behavior in counseling sessions. Both studies also used some form of model interpretation to understand how the models make predictions and what features or words characterize each class. Our study extends this line of research by using a different data set, coding scheme, and transformer model than the previous studies. Both related studies used the MISC codebook for data annotation and did not provide any estimates of coding reliability. In contrast, our study offers an in-depth account of the procedures and methodology, including reporting on coding reliability and fidelity measures. Studies that also used the MI-SCOPE (eg, Idalski Carcone et al [14]) used a small data set and obtained lower *F*_1_-scores than this study, highlighting the importance of using larger data sets to improve the performance of AI models in predicting MI behavior.

Our study contributes to the growing evidence base for MI as an effective intervention for various health-related behaviors. We showed that the AI models can accurately identify the effective ingredients of MI, such as client change talk and sustain talk, counselor affirmations, and reflection types—facilitating valuable counselor feedback. Furthermore, this study is the first to apply such a model to the domain of suicide prevention, which poses specific challenges and opportunities for MI. For example, counselors in a suicide prevention helpline need to adhere to MI but also balance building rapport; exploring ambivalence; focusing on engagement, collaboration, and empathy; and ensuring client safety.

### Strengths and Limitations

This study held several notable strengths. In our methodology, we adhered to the best AI practices. We used a holdout test set to evaluate the performance of the AI models, providing a realistic estimate of their generalization ability. Using diverse statistics to evaluate the model performances, we make the validation process comparable across all models. We clearly described the analytic strategy, ensuring transparency and reproducibility of the research process substantiated by the comprehensive supplemental material.

In the context of generalizability, classifying MI behavior could relatively easily be deployed in other domains and other languages. These days, large language models are being pretrained on many texts in multiple languages. From an AI point of view, implementing these methods in other online mental health helplines is relatively effortless.

While this study holds several strengths, there are also limitations. The data set used in this study is relatively small, which could lead to higher variance in the test set. Some MI code groups were underrepresented in the data set (eg, less evocative statements occurred in the text than nonevocative statements). This limited data set size may restrict the model’s ability to generalize to a broader range of MI conversations. It is important to note that the model’s performance in this specific domain of suicide prevention does not guarantee its effectiveness in other settings. To assess and train the model performances in another domain’s context, it is still necessary to gather domain-specific data.

Another limitation is that human experts coded the data set used for training and evaluation. While the expertise of human coders adds value, it is essential to acknowledge that the labeling process can still be subjective. Although not applicable to this study (since the intercoder reliability was sufficient, see section “Data Set Coding and Reliability”), individual coders may interpret and classify MI techniques and behaviors differently, potentially leading to inconsistent or inaccurately labeled data. This subjectivity in labeling could impact the performance of the automatic coding system and introduce errors or biases. A possible solution could be *active learning*—in which the AI model can interactively query a domain expert to label new data points with the desired outputs—or *reinforcement learning with human feedback* [38,39]. Reinforcement learning with human feedback has emerged as a powerful technique for refining these models. After initial training, the models receive feedback from human evaluators, enabling them to refine their approach.

A final limitation is the disregard for demographic traits of clients and counselors, such as age and cultural background. These characteristics could influence language use and model predictions. Further research is needed to refine the AI models with these factors, but these are not without ethical concerns [40]. In addition, years of counselor experience and MI proficiency could affect model effectiveness, with less experienced counselors likely benefiting more from the model.

### Implications for Clinical Practice

#### Leveraging AI Models for Clinical Support

There are several potential ways to incorporate AI models into clinical practice to enhance MI proficiency in online helplines for mental health:

By integrating AI models into chat-based counseling platforms, counselors can receive instant feedback on their MI behavior during sessions. This feedback allows counselors to review their generated messages before sending them and make necessary adjustments, such as changing a closed question to an open one. Initial results suggest that counselors find such systems acceptable [41], but more studies are needed to evaluate the reception and impact of these tools in different settings and populations.

By offering postsession feedback and training to counselors, they can reflect on their performance and pinpoint areas where they may require additional training or support. By analyzing data from multiple counseling sessions, AI models can detect patterns or trends in counselor MI behavior and develop tailored training programs that offer recommendations for training or support. This integration can assist counselors in identifying areas where they may need to modify their approach or apply MI techniques more effectively.

**Figure 2:**
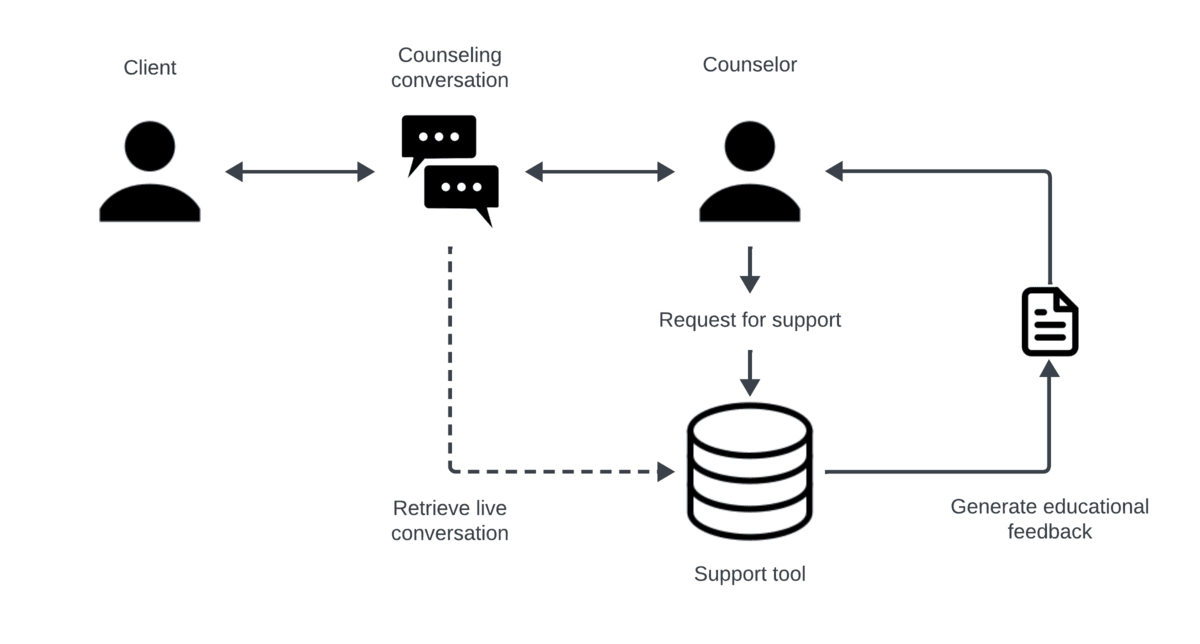
Schematic overview illustrating the proof of concept of the support tool.

Figure 2 shows a schematic overview illustrating the proof of concept of the support tool. Studies can investigate the feasibility of such a tool using a Wizard-of-Oz approach, where an experienced counselor acts as the support tool to simulate a best-case scenario. This setup could serve as a preliminary test for the viability of the support tool without requiring the development of a fully equipped AI tool for examining the potential advantages or disadvantages.

Madeira et al [42] proposed such a tool, and Salmi et al [41] examined it in focus groups with counselors and tested viability in a simulated environment. Studies emphasize the significance of tools that can help ease the workload of counseling [43,44]. Several studies also highlight relevant elements concerning the viability of AI in mental health counseling [43,45,46]. Therefore, a comprehensive evaluation of feasibility is necessary.

Helpline administrators or supervisors could also use AI models to monitor and evaluate the quality of counseling services provided by their organization. Using AI models to classify MI behavior and providing counselors with feedback offer a scalable and cost-effective solution for enhancing MI proficiency in helplines and other counseling settings. Many helplines struggle to find good staff, and the turnover is high, so reducing the time to proficiency is very valuable.

#### Evaluating the Effectiveness of AI Models in Clinical Practice

One way to evaluate the effectiveness of AI models in clinical practice would be to conduct pilot studies or randomized controlled trials analyzing changes in counseling outcomes over time. By comparing data from counseling sessions before and after the introduction of AI models, it would be possible to determine whether using these models leads to improved health outcomes for individuals seeking help.

Another way to evaluate the effectiveness of AI models would be to monitor changes in counselor MI behavior over time. By analyzing data from multiple counseling sessions, it would be possible to determine whether counselors who receive feedback from AI models improve their proficiency in applying MI techniques.

Feedback from counselors and clients could also provide valuable insights into the effectiveness of AI models in enhancing MI proficiency in chat-based counseling sessions. Counselors could provide feedback on the usefulness and accuracy of the provided feedback by the AI models, while clients could provide feedback on the quality of their interactions with counselors.

#### Next Steps to Take

Clinicians or researchers interested in leveraging AI for their specific use case in online helplines for mental health can already take initial steps to get started. A crucial first step in leveraging AI to enhance MI proficiency is to collect data from counseling sessions, such as chat transcripts and relevant metadata. Another important step is connecting with other clinicians and researchers in the field. By joining an active community of professionals working with MI and AI, one can benefit from the knowledge and resources created by others. These resources may encompass pretrained AI models, guidelines for collecting and analyzing data, and opportunities to collaborate and share knowledge with others in the field.

### Future Directions

While the results are promising, additional research is needed to evaluate the performance of these models on larger data sets with sufficient representation of each class. Future studies may explore alternative modeling techniques that better capture the conversational structure in classifying MI behavior. For instance, graph-based models can store information about the relationships between messages within and across conversations.

While this work and earlier research successfully quantified and validated the technical aspects of MI, it is also relevant to consider fundamental principles of MI. These principles contain the conversational processes that guide interactions between counselors and clients. Adhering to these processes ensures that counselors do not exceed the level a client is comfortable with and adapt their behavior appropriately based on the specific context. For instance, an appropriate question during the engaging process (building rapport between client and counselor) may become counterproductive during the evoking process, where the main goal is to elicit change talk. In these processes, concepts such as collaboration, engagement, and empathy also play a significant role. Recent work is already exploring integrating these concepts into AI models in the context of online helplines for mental health [44,47,48].

Furthermore, applying the methods used in this study to other languages and institutions could provide valuable additional validation of the study findings. A final point of future work would be to investigate and measure the potential improvement of MI quality in the chat helpline before and after counselors used MI insights during their conversations over time. This could also be combined with a support tool for MI feedback in a randomized controlled trial.

### Conclusions

The results of this study demonstrate that AI techniques can accurately classify MI behavior, indicating their potential as a valuable tool for enhancing MI proficiency in online helplines for mental health. Provided that the data set size is sufficiently large with enough training samples for each behavioral code, these methods can be trained and applied to other domains and languages, offering a scalable and cost-effective way to evaluate MI adherence, speed up behavioral coding, and provide therapists with personalized, quick, and objective feedback.

## Appendix

Multimedia Appendix 1 Detailed insights into our methods and findings.

## Abbreviations

AI: Artificial intelligence
AUC: area under the curve
BERT: Bidirectional Encoder Representations from Transformers
BERTje: monolingual Dutch BERT
LIWC: linguistic inquiry and word count
MI: motivational interviewing
MI-SCOPE: MI Sequential Code for Observing Process Exchanges
ML: machine learning
SHAP: Shapley Additive Explanations
SVM: support vector machine

## Data Availability

The data sets, materials, and code used or analyzed during this study are available from the corresponding author upon reasonable request.

## Data Transparency Statement

The data reported in this manuscript were collected as part of a larger data collection. Findings from the data collection have been reported in separate manuscripts. MS 1 [6] investigated the impact of training counselors in motivational interviewing on the outcomes of a chat-based suicide prevention helpline using the annotated MI behavioral codes and pre- and postchat questionnaire scores to measure suicidal ideation. MS 2 (this study) focuses on a subset of the collected data, including the raw text of the chat messages and their annotated MI behavioral codes to explore the use of automated behavioral coding to enhance the effectiveness of motivational interviewing. Unlike MS 1, MS 2 does not use suicidal ideation scores, and the raw text was used only for data annotation in MS 1 and not for analysis.
