# Supplementary material for: Automated Behavioral Coding to Enhance the Effectiveness of Motivational Interviewing in a Chat-Based Suicide Prevention Helpline: Secondary Analysis of a Clinical Trial
Source: J Med Internet Res. 2024 Aug 1;26:e53562. doi: 10.2196/53562 (PMC11327631; doi:10.2196/53562)
Supplement: Multimedia Appendix 1 [file jmir_v26i1e53562_app1.pdf]

Document Description. This supplementary material belongs to the article *"Automated Behavioral Coding to Enhance the Effectiveness of Motivational Interviewing in a Chat-Based Suicide Prevention Helpline: Secondary Analysis of a Clinical Trial."*

We give readers detailed insights into our methods and findings and describe them clearly and transparently, contributing to open science.

Multimedia Appendix 1

Related Work

Table S1

Schematic overview of related work that investigated automated coding of MI transcripts in counseling sessions using machine learning techniques.

Study	Application domain	Study size	Codebook	Best performing model	Coding reliability estimate
Hasan et al. (2019)	Weight loss	11,353 utterances<br>17 classes	MI-SCOPE	SVM.<br>0.75 accuracy	κ = 0.696
Carcone et al. (2019)	Weight loss	11,353 utterances<br>17 classes	MI-SCOPE	SVM.<br>0.66 F_1-score	κ = 0.696
Tanana et al. (2016)	Diverse settings<br>(Six MI clinical trials)	341 counseling sessions<br>175,000 utterances<br>17 classes	MISC	Multinomial regression.<br>Cohen's kappa varies per class from 0.20 to 0.95	Estimated κ = 0.713
Pérez-Rosas et al. (2017)	Several medical settings (smoking cessation, medication adherence, dietary changes, wellness coaching, medical encounters in dental practice, student counseling)	277 counseling sessions<br>22,719 utterances<br>7 classes	MITI	SVM.<br>Varying AUC scores per class up to 0.90	κ ranges from 0.28 to 0.64 among classes.<br>Estimated κ = 0.421
Tavabi et al. (2021)	Psychotherapy sessions with students having alcohol-related problems	219 counseling sessions<br>93,000 utterances<br>3 classes	MISC	Pre-trained RoBERTa.<br>0.66 F_1-score	Not reported
Saiyed et al. (2022)	Tobacco cessation	20,890 utterances<br>2 classes	MISC	RoBERTaGCN.<br>0.75 F_1-score	Not reported

Note. Cohen's kappa inter-rater reliability estimate is denoted by κ.

Feature Categories

Table S2

Overview of all feature categories, descriptions and corresponding feature sets.

	**Feature category** (# features)	**Description**	**Feature set**
1	Bag of Words (2,000)	Word occurrences in a chat message.	1
2	TF-IDF (2,000)	Relative importance of word occurrences across all chat messages.	1
3	Textual features (27)	Capturing a variety of textual information such as message length and the number of question marks.	2
4	Word embeddings (300)	Representing words as vectors of numbers in high-dimensional space to capture their semantic and contextual meaning.	3
5	Parts Of Speech (36)	Grammatical categories such as verbs, nouns, and prepositions.	4
6	Named Entities (18)	Real-world object categories (e.g., *Person, Location, Date*).	4
7	Dependencies (1,056)	Capture the grammatical structure of sentences by identifying relationships between the words.	4
8	Topics (42)	Identifying recurrent themes or topics.	5
9	Sentiment (29)	Extract emotions, appraisals, and attitudes toward different entities.	6
10	Cognitive Distortion Schemata (279)	Extracting language that indicates cognitive distortions (exaggerated or irrational thought patterns).	7
11	Temporal Patterns (63)	Capture the sequential message structure based on a temporal pattern mining algorithm.	8

Note. The hashtag character (#) means *'number of'*.

Classification Problems

Table S3

Number of classes for each classification problem, including train, validation, and test dataset size.

Classification problem	Number of classes	Number of instances		
		train	validation	test
**Counselor behavior**				
Fine-grained predictions	17	7,341	918	918
Evocative vs. non-evocative	2	7,341	918	918
MI-congruent vs. MI-incongruent	2	9,700	1,212	1,213
**Client behavior**				
Fine-grained predictions	4	9,485	1,186	1,186

Learning Algorithms

Table S4

Tried learning algorithms with varied parameters.

Learning algorithm	Hyperparameters
**Machine learning**	
Random Forest (RF)	Min. samples at leaf: [2, 10, 50, 100]<br>Split criterion: ['gini', 'entropy']<br>No. estimators: [10, 50, 100]
Decision Tree (DT)	Min. samples at leaf: [2, 10, 50, 100]<br>Split criterion: ['gini', 'entropy']
Support Vector Machines (SVM)	RBF kernel with coefficient γ: [1e-1, 1e-2]<br>Regularization parameter C: [1, 10, 100]
k-Nearest Neighbors (kNN)	Minkowski distance metric with number of neighbors: [1, 2, 5, 10]
**Transfer learning**	
BERTje finetuned	Learning rate: 2e-5<br>No. Epochs: 10<br>Optimizer: AdamW<br>Max token count: 256<br>Batch size: 32<br>Criterion: BCEloss<br>Activation function: Sigmoid

Evaluation Metrics

Confusion Matrix. A confusion matrix is a specific $N \times N$ table layout (where N is the number of classes) that allows visualization of the performance of an algorithm. Each row of the matrix represents the instances in an actual class, while each column represents the instances in a predicted class. An example of a confusion matrix is shown in Figure S1. A confusion matrix allows for the computation of different evaluation metrics, such as *accuracy, precision, and recall.*

Figure S1

Example Confusion Matrix.

		**Predicted outcome**	
		**Class A**	**Class B**
**Actual value**	**Class A**	True Positives (TP)	False Negatives (FN)
	**Class B**	False Positives (FP)	True Negatives (TN)

Accuracy. The accuracy of a machine learning classifier is the fraction of correct predictions (Equation 1).

$$Accuracy = \frac{(TP + TN)}{(TP + TN + FP + FN)} \tag{1}$$

Precision. Equation 2 shows the formula for computing the precision of a classifier. Precision is intuitively the ability of a classifier not to label a negative instance as positive. The best value is 1, and the lowest value is 0.

$$Precision = \frac{TP}{TP + FN} \tag{2}$$

Recall. Equation 3 shows the formula for computing the recall of a classifier, which is the classifier's ability to find all positive samples. A value of 1 is the best, while 0 is the lowest.

$$Precision = \frac{TP}{TP + FN} \tag{3}$$

F1 Score. The F_1 score (Equation 4) is the harmonic mean of precision and recall. It ranges from 0 to 1, with 1 being the best value and 0 being the worst. The F_1 score is a better evaluation metric for classifiers with unbalanced class distributions because it minimizes the false positives and negatives and seeks a balance between precision and recall. Considering a multi-class classification problem, one could compute the micro and macro average F_1. The macro-average calculates the metric for each class independently and then takes the mean, giving equal weight to all label classes. A micro-average aggregates the contributions of all classes to compute the average metric, taking class imbalance into account. Another possibility is to treat classification as a multi-label classification problem, where the classifier returns a probability distribution over all classes

for each instance. In this case, the *sample average* F_1 could be computed by calculating the F_1 score for each sample and returning the average.

$$F_1 = 2 * \frac{(precision \times recall)}{(precision + recall)} \tag{4}$$

AUC-ROC. When one needs to evaluate or visualize the performance of a multi-class classification problem, the AUC (Area Under the Curve) - ROC (Receiver Operating Characteristics) curve is a convenient tool (Figure S2). They can provide a richer measure of classification performance than scalar measures such as accuracy. The AUC - ROC curve is a performance measurement for classification problems at various threshold settings. The ROC is a probability curve, and the AUC represents the degree or measure of separability. It tells how much the classifier is capable of distinguishing between classes. The True Positive Rate (TPR) against the False Positive Rate (FPR) presents the ROC curve, where the TPR appears on the y-axis and the FPR on the x-axis. The higher the AUC, the better the model predicts all true positives correctly. An ideal classifier will have a ROC where the graph would hit a True Positive Rate of 100% with zero false positives. For example, when the AUC is 0.7, it indicates a 70% likelihood that the classifier can differentiate between positive and negative classes.

Figure S2

Example AUC - ROC Curve.

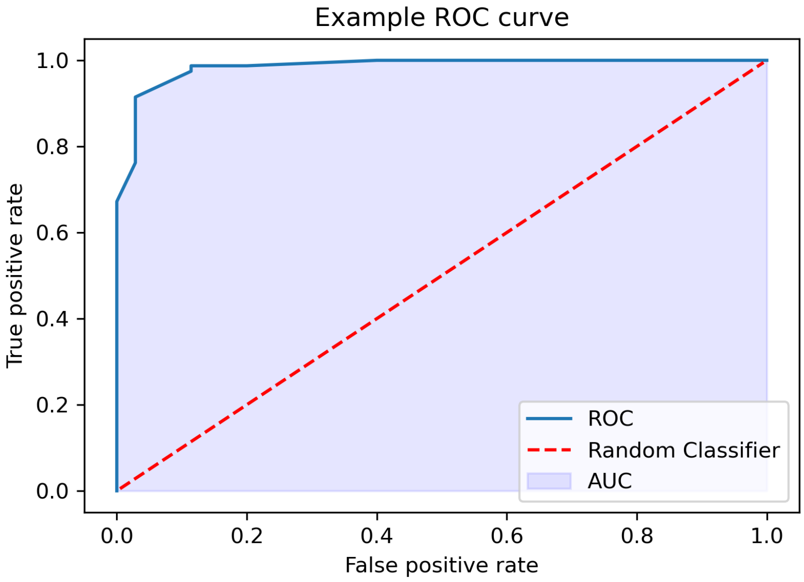

In the case of multi-class classification, one can use the *One-vs-Rest* methodology to plot N AUC-ROC curves, where N is the number of classes. For instance, given three class labels (A, B, and C), one could plot a curve for class A against B and C, another for class B against A and C, and the third for class C against A and B. Moreover, one could compute the micro and macro-average AUC with the same idea as with the F1 score; the micro-average AUC is the weighted-average AUC score (it takes class imbalance into account), and the macro-average AUC is simply the average of the AUC scores for all classes.

Cohen's Kappa. The Kappa statistic expresses the level of agreement between two annotators on a classification problem (Cohen, 1960). It is defined as given in Equation 5.

$$\kappa = (p_o - p_e)/(1 - p_e) \tag{5}$$

p_o represents the empirical probability of agreement on the label assigned to any sample (the observed agreement ratio), and p_e is the expected agreement when both annotators assign class labels randomly. p_e is estimated using a per-annotator empirical prior over the class labels (Artstein & Poesio, 2008). The kappa statistic is a number between -1 and 1. The maximum value means complete agreement; zero or lower means chance agreement.

Machine Learning Classification Performances

Counselor Behavior

Table S5

Machine learning algorithm performances on different feature subsets for predicting counselor behavior.

	**DT**			**RF**			**SVM**			**kNN**		
	Micro avg AUC	*Macro avg AUC*	*Sample avg F_1*	*Micro avg AUC*	*Macro avg AUC*	*Sample avg F_1*	*Micro avg AUC*	*Macro avg AUC*	*Sample avg F_1*	*Micro avg AUC*	*Macro avg AUC*	*Sample avg F_1*
**Feature set**												
Feature subset 1	0.80	0.75	0.43	0.92	0.91	0.56	0.94	0.93	0.60	0.68	0.66	0.40
Feature subset 2	0.85	0.81	0.52	0.94	0.93	0.58	0.94	0.93	0.60	0.68	0.65	0.40
Feature subset 3	0.82	0.77	0.49	0.92	0.91	0.53	**0.95**	**0.94**	**0.63**	0.86	0.82	0.48
Feature subset 4	0.82	0.77	0.49	0.92	0.91	0.53	0.94	0.93	0.63	0.86	0.81	0.48
Feature subset 5	0.82	0.77	0.49	0.92	0.91	0.53	0.94	0.93	0.63	0.86	0.81	0.48
Feature subset 6	0.82	0.78	0.51	0.92	0.91	0.54	0.94	0.93	0.62	0.87	0.82	0.50
Feature subset 7	0.83	0.79	0.51	0.92	0.91	0.52	0.94	0.93	0.62	0.87	0.81	0.50
All features	0.89	0.84	0.51	0.93	0.91	0.53	0.91	0.88	0.53	0.82	0.76	0.42

Client Behavior

Table S6

Machine learning algorithm performances on different feature subsets for predicting client behavior.

	**DT**			**RF**			**SVM**			**kNN**		
	Micro avg AUC	*Macro avg AUC*	*Sample avg F_1*	*Micro avg AUC*	*Macro avg AUC*	*Sample avg F_1*	*Micro avg AUC*	*Macro avg AUC*	*Sample avg F_1*	*Micro avg AUC*	*Macro avg AUC*	*Sample avg F_1*
**Feature set**												
Feature subset 1	0.79	0.70	0.55	0.84	0.80	0.60	0.82	0.79	0.59	0.73	0.66	0.50
Feature subset 2	0.80	0.75	0.57	0.84	0.82	0.59	0.81	0.79	0.56	0.75	0.71	0.51
Feature subset 3	0.80	0.74	0.56	0.83	0.81	0.56	0.84	0.82	0.61	0.80	0.74	0.59
Feature subset 4	0.80	0.74	0.56	0.83	0.81	0.56	0.84	0.82	0.61	0.80	0.73	0.58
Feature subset 5	0.80	0.74	0.56	0.83	0.81	0.56	0.84	0.82	0.61	0.81	0.73	0.58
Feature subset 6	0.83	0.79	0.60	0.84	0.83	0.58	0.85	0.83	0.64	0.83	0.78	0.62
Feature subset 7	0.83	0.79	0.60	0.84	0.82	0.58	**0.86**	**0.83**	**0.65**	0.83	0.78	0.62
All features	0.84	0.79	0.62	0.85	0.83	0.60	0.85	0.83	0.63	0.81	0.76	0.60

Feature Contributions

Table S7

Most influential features and word combinations contributing to the prediction outcomes and language character per class for counselor- and client behavior.

Class	Highest feature importance	Most occurring word combinations
**Counselor behavior**		
Advise with Permission (AWP)	# lowercase letters, # vowels	*seeking distraction; own environment; I think that; seeking contact; thoughts; express emotion, pleasant manner; creative; sports; general practitioner*
Advise without Permission (ADW)	# question marks	*I think that; how/what about; maybe it is good to; try to hold on; seek distraction; let it sink in; in any case; call 911 (Dutch: 112)*
Affirm (Aff)	positive sentiment, subjectivity score	*good for you; very wise of you; how great; seems like a good idea; good to hear*
Closed Question	# question marks	*did I get that right; do you ever; do you think that; do you also have; is this something to; are you still there; would you manage to; does your therapist know*
Confront (Con)	# question marks, neutral sentiment	*after hearing you; I think you; sounds like; I can imagine; a long time; crisis service; suicidal thoughts*
Emphasize Control (Econ)	use of pronouns	*what would you like to discuss; what do you need the most; look together; a friendly and listening ear; is there still something else*
Filler (Fill)	# stopwords,[a] sentence length	*welcome to the chat; thank you for waiting; thank you for your openness; you're welcome; no problem; you too; okay; hmm*
General Information (GI)	use of punctuation, # special characters	*online therapy; regular psychologist; website; via email; five working days; finding information; registration; https://www.113.nl*
Open Question (OQ+)	# question marks, positive sentiment	*what would you need; what do you like to do; what could it bring you; what do you think of . . . ; how would you; what do you usually do*
Open Question (OQ−)	# question marks, negative sentiment	*what happened; how come; what makes you think that; what's going on; what is the worst that could happen; what can you tell more about . . .*
Open Question (OQ0)	# question marks, use of adjectives	*how does this feel for you; what is your point of view about; how would it be like to . . . ; what do you think; what makes that; how would you*

Continued on next page

Table S7 – *Continued from previous page*

Class	Highest feature importance	Most occurring word combinations
Permission Seeking (Perm)	use of the word "I", # unique words	*shall we discuss our ideas together; is it okay for you if; are you comfortable with this; is it an idea to; share information*
Reflection (+)	use of the word "you", positive sentiment	*sounds like; you indicated that; you're describing; you feel; if I understand correctly; on one side; on the other side; conflicted; listening ear; look together; for now you want*
Reflection (0−)	use of the word "I", negative sentiment	*you feel drained; clearly, there's a lot going on; you've had some negative encounters; gone through a bad time; it feels like; suffering from suicidal thoughts; tension; restlessness*
Self Disclose (Sdis)	use of the word "I"	*from my own experience; I know; I see that you; I think; I hope you; for me; I am; I find it; oh sorry*
Structure (Str)	# question marks	*hi, you are speaking with . . . ; just a moment; I'll be right back to you; close the chat; read back our conversation*
Support (Sup)	neutral sentiment	*sorry to hear; sad to hear this; I understand your thoughts; that does sound like; I can imagine; good luck; get well soon*
**Client behavior**		
Ask (Ask)	# question marks	*what do you mean by that; what can I do; what should I; but how can I; what if; do you agree with; what kind of help*
Change Talk (X Csa+)	negative sentiment, negations	*good idea; very nice; I could try that; I think so; will help; talk about it; look for a distraction; listen to music; watch TV*
Follow/Neutral (FN)	# short words[b]	*that's fine; I don't know if; nothing to worry about; I know; thanks for your time / help; yes; no; thanks for the conversation*
Sustain Talk (X Csa−)	negative sentiment, negations	*I don't want to; I'm afraid; when I'm not here anymore; I don't know how; I find it difficult; I feel really bad*

Note. The hashtag character (#) means *"number of"*.
[a]Stopwords: commonly used words in a language (such as "the", "a", "an", "in" in English).
[b]Short words: words with less than five characters.
